# Molecular Simulations of Biomembranes: From Biophysics Fundamentals to Biological Function

**DOI:** 10.3390/membranes16030080

**Published:** 2026-02-24

**Authors:** Md. Ashrafuzzaman, Jack A. Tuszynski

**Affiliations:** 1 Doping Research Chair, Deanship of Scientific Research, King Saud University, Riyadh 11451, Saudi Arabia; 2 Department of Physics, University of Alberta, Edmonton, AB T6G 1Z2, Canada; jackt@ualberta.ca; 3 Department of Mechanical and Aerospace Engineering, Politecnico di Torino, 10126 Torino, Italy

Biomembrane transport mechanisms [1,2] are linked to various physical phenomena governed by physics laws and principles. The physicochemical properties of the key membrane components, such as phospholipids, cholesterol, and membrane proteins including ion channels, glycocalyx, hydrocarbons, etc., play fundamental roles in constructing the membrane’s physical structures, maintaining its structural stability and dynamical nature [3], and helping it to continuously be active in safeguarding the transmembrane transport phenomena [4,5] in order to support cellular homeostasis. To address these complex topics, we launched a Special Issue and invited experts from around the globe to contribute articles. We finalized six of them, which readers may find useful for enhancing their fundamental knowledge of this fascinating area of cell biology, in addition to enriching their understanding of specific topics.

Malfunctions of the transport properties [6,7] of plasma, mitochondrial, and nuclear membranes are intricately linked to the development of diseases, including cancer, Alzheimer’s disease as well as many other neurodegenerative diseases, and other pathologies. The identification of specific pharmacological agents that might play therapeutic roles [8,9] in the development of successful therapies in the future is crucial for medical science. Transmembrane drug delivery is another important issue that we encounter while designing and optimizing the delivery of drug molecules into sites of target biomolecules (proteins, nucleic acids, etc.) that are linked to specific diseases or structural disorders inside cells. This Special Issue has accumulated articles to address the roles of membranes in both therapeutic and drug delivery aspects of medical research using the membrane-associated biophysical actions of specific molecules.

Understanding the structural composition and associated physical phase properties of a membrane [10,11] can be achieved by simulating membranes utilizing various lipid composition models as well as incorporating external agents that may be accommodated inside the membrane’s hydrophobic environment. The structural integrity of a membrane is not susceptible to alterations in electric charge properties [12,13] in the vicinity. Simulation-based [14] and experimental [15,16] investigations addressing these issues have led to the discovery of conditions and pharmacological agents that are important in developing successful therapeutics. This Special Issue has accumulated articles to aid in this area of applied medical research.

Overall, although this Special Issue is relatively small (see the “List of Contributions”), it has accumulated a few important articles to address key biomembrane properties and phenomena that are not only important in understanding the roles of cell membranes in cell functions linked with major cell signaling pathways, but also aid in the development of strategies through biomedical research in drug discovery and delivery. Following this Special Issue, subsequent volumes may be released with a view to elaborating this area with up-to-date scientific knowledge to foster both fundamental and applied medical research.
